# Investigator analytic repeatability of two new intervertebral motion biomarkers for chronic, nonspecific low back pain in a cohort of healthy controls

**DOI:** 10.1186/s12998-020-00350-5

**Published:** 2020-11-24

**Authors:** Daphne To, Alexander Breen, Alan Breen, Silvano Mior, Samuel J. Howarth

**Affiliations:** 1 Canadian Memorial Chiropractic College, 6100 Leslie Street, Toronto, Ontario M2H 3J1 Canada; 2 Centre for Biomechanics Research, AECC University College, Parkwood Campus, Parkwood Road, Bournemouth, Dorset BH5 2DF UK

**Keywords:** Back pain, Biomarkers, Kinematics, Fluoroscopy, Repeatability

## Abstract

**Background:**

Understanding the mechanisms underlying chronic, nonspecific low back pain (CNSLBP) is essential to advance personalized care and identify the most appropriate intervention. Recently, two intervertebral motion biomarkers termed “Motion Sharing Inequality” (MSI) and “Motion Sharing Variability” (MSV) have been identified for CNSLBP using quantitative fluoroscopy (QF). The aim of this study was to conduct intra- and inter-investigator analytic repeatability studies to determine the extent to which investigator error affects their measurement in clinical studies.

**Methods:**

A cross-sectional cohort study was conducted using the image sequences of 30 healthy controls who received QF screening during passive recumbent flexion motion. Two independent investigators analysed the image sequences for MSI and MSV from October to November 2018. Intra and inter- investigator repeatability studies were performed using intraclass correlations (ICC), standard errors of measurement (SEM) and minimal differences (MD).

**Results:**

Intra-investigator ICCs were 0.90 (0.81,0.95) (SEM 0.029) and 0.78 (0.59,0.89) (SEM 0.020) for MSI and MSV, respectively. Inter-investigator ICCs 0.93 (0.86,0.97) (SEM 0.024) and 0.55 (0.24,0.75) (SEM 0.024). SEMs for MSI and MSV were approximately 10 and 30% of their group means respectively. The MDs for MSI for intra- and inter-investigator repeatability were 0.079 and 0.067, respectively and for MSV 0.055 and 0.067.

**Conclusions:**

MSI demonstrated substantial intra- and inter-investigator repeatability, suggesting that investigator input has a minimal influence on its measurement. MSV demonstrated moderate intra-investigator reliability and fair inter-investigator repeatability. Confirmation in patients with CNSLBP is now required.

## Background

The massive societal burden of chronic pain has prompted calls for urgent development of validated biomarkers to facilitate mechanism-based management as an advance over current risk-based approaches [1]. A number of biomarkers have been suggested for chronic nonspecific low back pain (CNSLBP), but few have been fully validated [2].

A biomarker is an objectively measurable variable that correlates with the presence of a condition, making it possible to seek other related variables that may support a diagnostic approach based on mechanisms [3]. Biomechanical variables based on intervertebral motion have been explored as potential biomarkers for CNSLBP and the emergence of multilevel continuous dynamic imaging systems in place of static ones has produced an improved gold standard for intervertebral motion measurement [4].

Recently, intervertebral motion biomarkers based on the sharing of angular displacements between levels during recumbent lumbar flexion as measured using quantitative fluoroscopy (QF) have been identified for CNSLBP and their presence has been confirmed by replication studies. These biomarkers have been termed Motion Sharing Inequality (MSI) and Variability (MSV) [5–7], however, the evaluation of these measurements is incomplete. Although the repeatability and accuracy of the measurement of individual level angular motion have been established and the intrasubject repeatability (or measurement error) of the multiple level measures of MSI and MSV has recently been determined, the analytical intra- and inter-investigator errors remain unknown [7–10]. However, the instrument error has been previously addressed [11].

These errors refer both to the extent to which two measurements, obtained from the same image sequence by two separate investigators agree with each other (agreement) and to which measured objects can be distinguished from each other (reliability) [12]. Without the former, the capacity to correlate the strength of a back pain biomarker with its underlying mechanisms (such as passive tissue compromise) and interventions (such as manual therapies), is weakened, thus diminishing its value. In these scenarios, investigators would be less able to use the biomarkers to mechanistically develop therapies, as the two are intricately related [1]. Therefore, in order for further studies on the role of MSI and MSV in CNSLBP to be performed, it is important to undertake intra- and inter-investigator repeatability studies to determine the extent to which observer error affects their measurement. Thus, the aim of our study was to determine the intra-and inter-investigator analytical repeatability for the intervertebral motion sharing parameters, MSI and MSV, in a healthy population using QF as evidence of its construct validity with a lower confidence limit of the ICCs being > 0.6 as evidence of at least moderate reliability.

## Methods

### Study design

We performed a cross-sectional cohort study from October to November 2018 to assess intervertebral motion sharing in the lumbar spine using fluoroscopic image sequences previously obtained according to a standardised recumbent protocol for the purpose of building a normative database [13].

### Participants

A random sample of 30 QF image sequences was obtained from a database of 101 healthy control volunteers aged between 10 and 70 years who were recruited from students and visitors to the AECC University College. To be included, participants had to have a body mass index of less than 30, no medical radiation exposure of > 8 mSv in the previous 2 years, no pregnancy (females) and no back pain that limited their normal activity for more than 1 day in the previous year.

All participants gave informed consent. The original study received ethical approval from the UK National Research Ethics Service (South West 3, REC reference 10/H0–106/65). Data handling, processing and analysis procedures for the current study were approved by the research ethics board at the Canadian Memorial Chiropractic College (REB approval #1807X01).

### Instrumentation

The image sequences were collected using a Siemens Arcadis Avantic digital C-arm fluoroscope (VC10A, Siemens AG, Erlagen, Germany) at 15 Hz. Exposure factors were determined by an automatic exposure device.

### Image acquisition

**Fig. 1:**
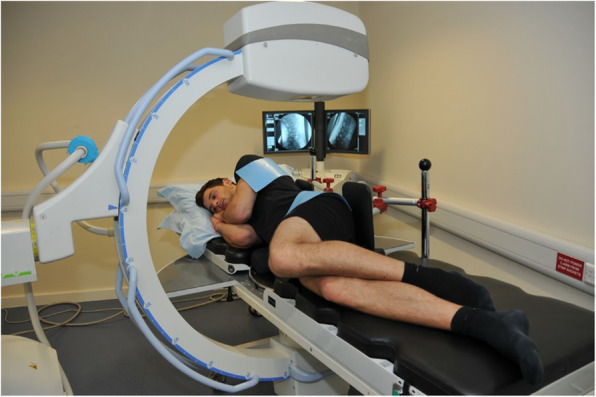
Apparatus for passive recumbent lumbar spine quantitative fluoroscopy image acquisition

Procedures for image acquisition for passive recumbent lumbar spine flexion and return have been previously described by Breen and Breen [5]. Briefly, participants were positioned, unrestrained, on their side on an articulated table (Atlas Clinical Ltd., Lichfield, UK) where the trunk segment of the table was motorised and driven by a controller (Fig. 1). Lead shielding was placed over the thyroid, breasts, and gonads at all times during image acquisition. The digital fluoroscope was positioned with its central ray aligned through the intervertebral disc between the third and fourth lumbar vertebrae (L3-L4). This was further aligned with the centre of rotation of the trunk segment of the table to provide the best chance that the imposed flexion movement would be located at the L2-S1 spinal levels. Fluoroscopy was synchronised to the motion of the table. This facilitated imaging from the second lumbar (L2) to the first sacral (S1) vertebra. The motorised table accelerated at 6^o^/s^2^ for the first second followed by a uniform velocity of 6^o^/s for the remainder of the motion until a maximum forward flexion angle of 40^o^ between the trunk and lower body was obtained. It then decelerated at the same rate in the final second of the outward motion, followed by the return motion which mirrored the outward kinematics.

### Image analysis

**Fig. 2:**
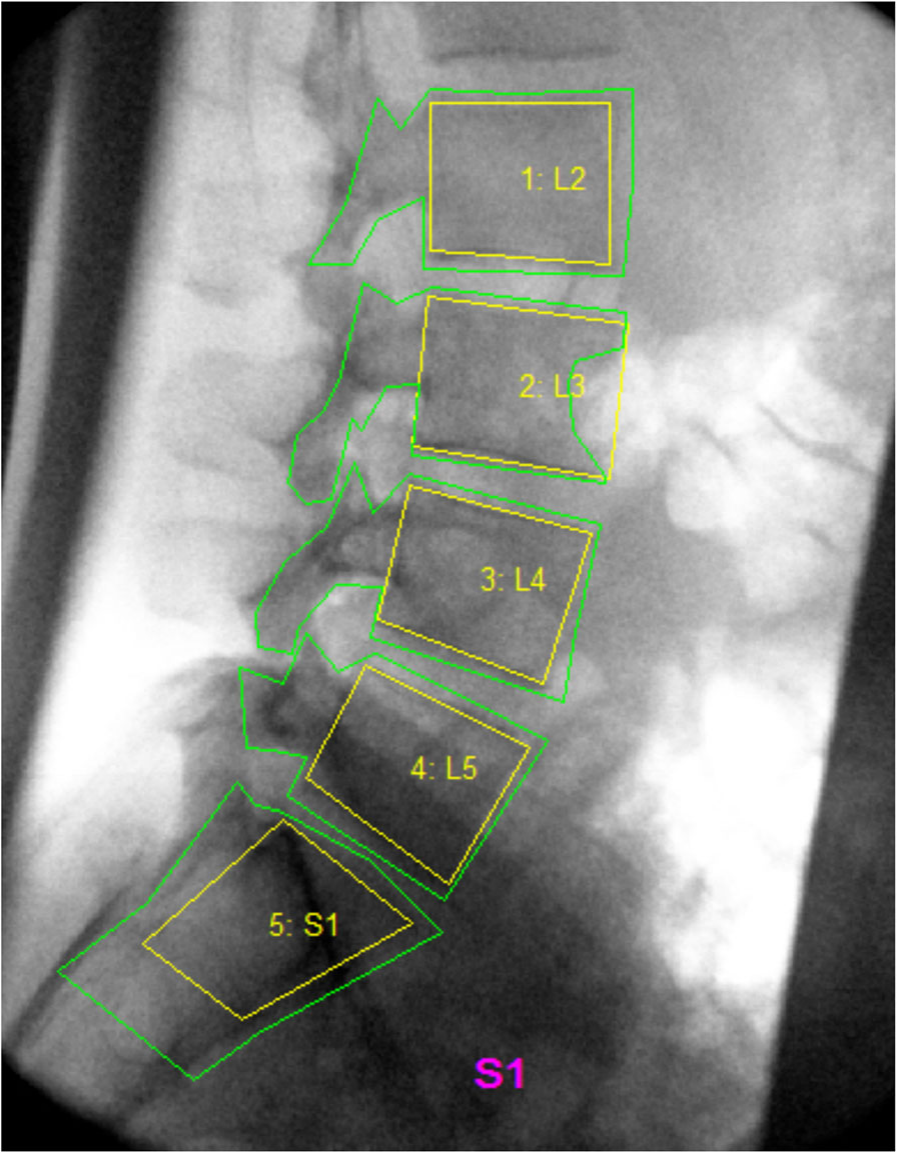
Reference templates (yellow) and tracking templates (green) were created on the first image of each sequence to allow for automated frame-to-frame tracking of the vertebral bodies in subsequent images of the sequence

The image sequences were anonymised, exported to a computer workstation, and analysed using manual first image registration followed by frame-to-frame tracking [13] using codes written in Matlab (V2013 – The MathWorks Inc., Natick, Massachusetts, USA). All images in each sequence underwent investigator-defined edge enhancement. This specifically assisted with first image registration that required the creation of reference and tracking templates. Reference templates were created by the investigator manually marking the corners of each visible vertebral body on the first image of each sequence. These were used to construct the geometric positions of the vertebrae as the selection of vertebral body corners could not systematically bias the outputs of the analysis. The investigator also created tracking templates on the first image of each sequence by placing cursor lines around each vertebral body (Fig. 2). These tracked the vertebral body outlines and measured their frame to frame displacements. First image registration was repeated five times to facilitate automated frame-to-frame tracking of the vertebral bodies in subsequent images of the sequence. The reference and tracking templates were linked in order to verify tracking and calculate intervertebral rotations at each image in a sequence [7, 13]. Tracking throughout the entire motion sequence was verified by the investigator by visually inspecting all image sequences with video playback and repeating image registration for any tracking that failed [7]. On average, one test per level per sequence had to be re-tracked.

### Repeatability study

To assess inter-investigator repeatability, two investigators (AxB and DT1) independently performed first image registration for each of the anonymised image sequences. To assess intra-investigator repeatability, one investigator performed first image registration for all 30 image sequences on a second occasion (DT2) that occurred at least 1 week after their first attempt. The anonymised image sequences were presented in different random orders during analysis.

### Data processing and analysis

**Fig. 3:**
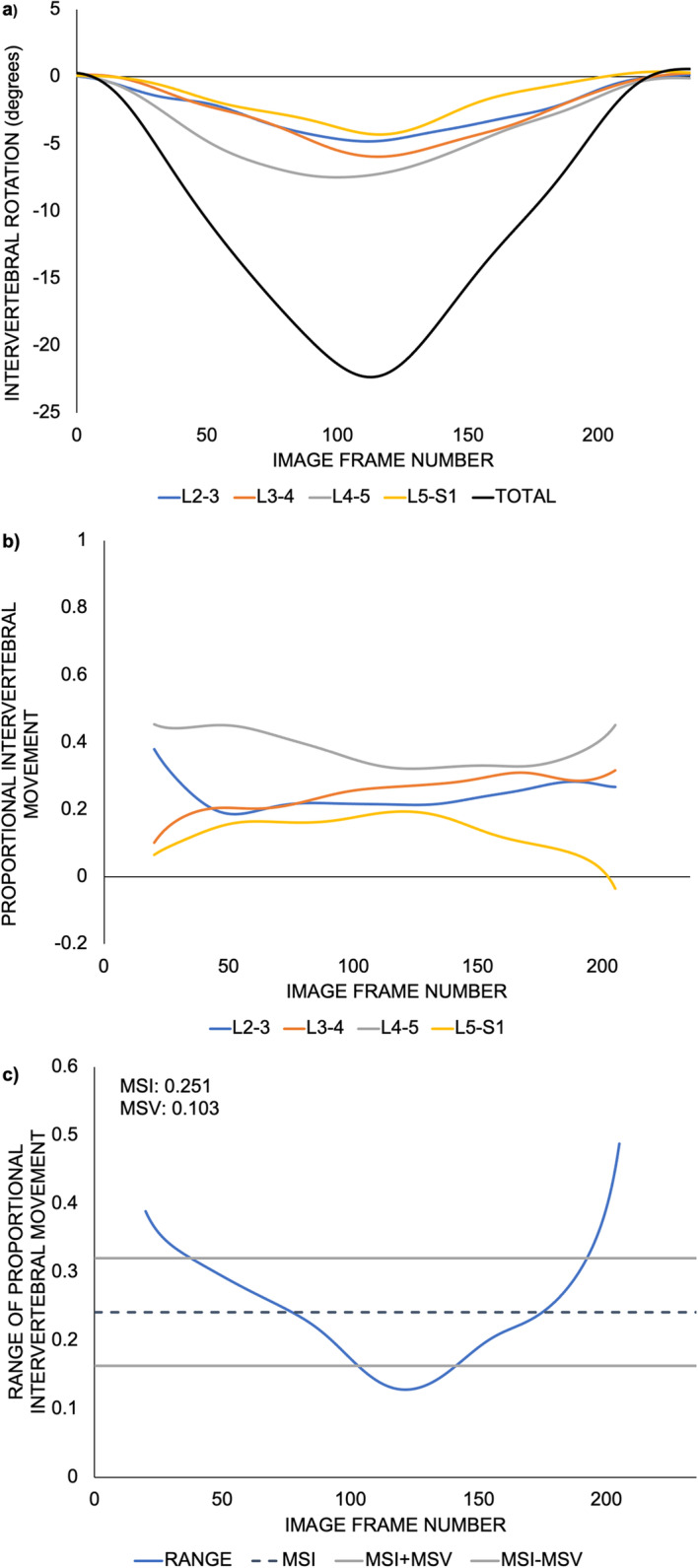
Derivation of motion sharing inequality (MSI) and motion sharing variability (MSV) from a representative QF image sequence obtained from one participant during lumbar flexion and return. Absolute intervertebral rotations, where the forward flexion direction is considered a decrease in intervertebral angle (**a**), are transformed into proportional intervertebral rotations (**b**), which allow for the calculation of the ranges of the proportional intervertebral movement. MSI is the average of the range of proportional intervertebral movement, while MSV is the standard deviation of the range of proportional intervertebral movement (**c**)

Changes in intervertebral angular position from the initial position during forward flexion and return of the identified joints from L2-L3 to L5-S1 were calculated throughout each motion sequence (Fig. 3a). Intervertebral angles were proportionately scaled as a ratio of the overall lumbar spine angle from L2 to S1 (Fig. 3b). Changes in intervertebral angle from the participants’ starting position are small at the beginning and end of their bending sequences, thus, these data points are close to the precision limit of the QF system (0.52°) [8]. Therefore, only the middle 80% of movement was considered for analysis to remove error amplification during the initial and final parts of movement [6, 14]. The range of proportional intervertebral movement was calculated for each image in the sequence (Fig. 3c) [5]. MSI, a measure of the inequality of passive restraint, was calculated as the average of the range of proportional intervertebral movement (fRC_i_) across the (N) images of the motion sequence (Fig. 3c) [5]:

$$ \mathrm{MSI}=\frac{\sum_{\mathrm{i}=1}^{\mathrm{N}}\mathrm{fR}{\mathrm{C}}_{\mathrm{i}}}{\mathrm{N}} $$

MSV, a measure of the unevenness of control, was calculated as the standard deviation of the range of proportional intervertebral movement across the image data points of the motion sequence (Fig. 3c) [5]:

$$ \mathrm{MSV}=\sqrt{\frac{\sum_{\mathrm{i}=1}^{\mathrm{N}}{\left(\mathrm{fR}{\mathrm{C}}_{\mathrm{i}}-\mathrm{MSI}\right)}^2}{\mathrm{N}}} $$

### Statistical analysis

Statistical analyses were performed in R [15, 16]. Three estimates of the group descriptive measures (means and standard deviations) were determined for each of MSI and MSV (DT1, DT2 and AxB). Estimates of intra- and inter-investigator reliability for MSI and MSV were determined using intraclass correlation coefficients (ICCs) using a single measures, two-way random-effects model [17]. The 95% confidence interval (95% CI) limits for these ICCs were also determined. The ICCs were categorised qualitatively as slight (0.11–0.40), fair (0.41–0.60), moderate (0.61–0.80), and substantial (0.81–1.00). ICCs and the appropriate pooled standard deviations were used to determine standard errors of measurement (SEMs), calculated as the root of the error variance from the two-way, random effects ANOVA models and minimal differences (MDs), calculated as SEM × 1.96 × √2 [18].

## Results

### Participant demographics

QF image sequences from 30 healthy participants (15 male, 15 female) were analysed. The mean age of participants was 35 (SD 14, range = 22–65). The mean body mass index was 23.5 kg/m2 (SD 3.2, range = 16.9–28.2 kg/m^2^). The mean effective radiation dosage was 0.18 mSv (SD 0.03, range = 0.12–0.25 mSv).

### Repeatability of motion sharing

**Fig. 4:**
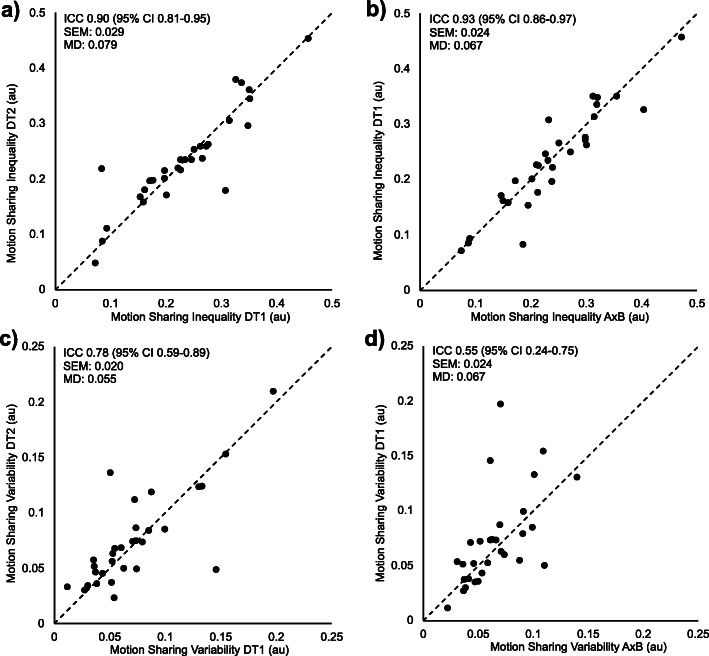
Scatterplots and intraclass correlation coefficients (ICCs) for **a** intra-investigator repeatability for motion sharing inequality (MSI), **b** inter-investigator repeatability for MSI, **c** intra-investigator repeatability for motion sharing variability (MSV), and **d** inter-investigator repeatability for MSV with standard errors of measurement (SEMs) and minimal differences (MDs). The dashed line represents the line of identity between observations (**a** and **c**) or investigators (**b** and **d**)

Group means and standard deviations for MSI and MSV for all investigators are reported in Table 1. Intra- and inter-investigator reliability were substantial for MSI (0.90, 95% CI 0.81–0.95 and 0.93, 95% CI 0.86–0.97, respectively) (Fig. 4). Intra-investigator reliability (0.78, 95% CI 0.59–0.89) was moderate for MSV and inter-investigator reliability was fair (0.55, 95% CI 0.24–0.75). The SEM, expressed also as a percentage of the group means for MSI, for intra- and inter-investigator repeatability was 0.029 (12%) and 0.024 (10%), respectively. The MD for MSI for intra- and inter-investigator repeatability was 0.079 and 0.067, respectively. The SEM, expressed also as a percentage of the group means for MSV, for intra- and inter-investigator repeatability was 0.020 (27%) and 0.024 (35%), respectively. The MD for MSV for intra- and inter-investigator repeatability was 0.055 and 0.067, respectively. For completeness, the ICC’s, SEMs and MDs were also calculated between the AxB and DT2 observations. No notable difference between observer combinations were found.

**Table 1:** Group means and standard deviations for MSI and MSV for all investigators (investigator 1 and investigator 2 at two time points)

	MSI		MSV	
	Mean	Standard Deviation	Mean	Standard Deviation
Investigator 1 (AxB)	0.24	0.09	0.07	0.03
Investigator 2 Time 1 (DT1)	0.23	0.09	0.07	0.04
Investigator 2 Time 2 (DT2)	0.24	0.09	0.08	0.04

## Discussion

Understanding the mechanisms underlying back pain can support personalized care beyond risk-based management [19]. Such an understanding can assist in selecting the appropriate care, which may have varying effects. For example, manual therapies are widely regarded as having both biomechanical and neurophysiological effects [20]. Thus, identifying biomarkers for back pain can support methods for appropriate treatment selection.

Intervertebral motion sharing inequality and motion sharing variability measured using QF image sequences have been hypothesised to be possible biomarkers for mechanical causes of pain in patients with CNSLBP [5, 6]. Establishment of measurement properties such as reliability and validity are necessary for determining the utility of QF measures as biomarkers [21]. In particular, for measurements such as MSI and MSV, it is imperative that the necessary investigator input to derive the measures does not introduce substantial variability in the actual measurements. For QF, the investigator is required to provide input to initiate image analysis, image processing, and the quantification of intervertebral motion. As such, the purpose of the current investigation was to establish intra- and inter-investigator repeatability, particularly associated with investigator input, for intervertebral motion sharing (MSI and MSV). The results from our study suggest that investigator input had minimal impact on MSI and a greater impact on MSV for image sequences obtained in a healthy population during passive recumbent lumbar spine flexion.

Two sources of systematic and random error in QF that may affect the measurements of intervertebral motion sharing are trial-to-trial variability within a subject (intrasubject variability) and error from investigator input (intra- and inter-investigator variability). A recent study established intrasubject reliability for MSI and MSV in passive recumbent and active weight-bearing lumbar spine flexion, extension, and lateral bending and another study determined the machine error for single level motion [10, 11]. Other previous work in passive recumbent flexion reported intrasubject reliability (which includes instrument error) as substantial for MSI (ICC 0.61, 95% CI 0.34–0.78) and moderate for MSV (ICC 0.41, 95% CI 0.00–0.66). The minimal detectable change was reported as 0.31 for MSI and 0.12 for MSV. Our findings suggest that the reported ICCs and minimal detectable changes are subject to the intra- and inter-investigator variability as well as trial-to-trial variability. Given that an investigator is highly involved in the process of image acquisition, image analysis, and data processing, other sources of variability may be introduced. These sources of variability also include instrument measurement error and trial-to-trial variability of the subject’s positioning during image acquisition and/or the investigator marking of the image sequences.

The likelihood of setup error, positioning error or exposure error is minimal as this would be immediately apparent from inspection of the image sequences after screening and would require a second exposure. If dose reference levels were likely to be exceeded, the investigation would be abandoned. Thus, only accredited operators are permitted to perform QF acquisitions, avoiding this outcome.

The current study controlled for intrasubject variability by using the same set of image sequences from each participant for image analysis, allowing for the analysis of error associated with investigator input. MSI and MSV are derived from intervertebral rotations; however, existing reliability estimates for intervertebral rotations are inadequate for estimating the reliability for MSI and MSV. Intervertebral rotations are determined for each level, but MSI and MSV are determined for all of the levels combined and are derived from proportional intervertebral movement. Our study’s results demonstrated that the intra- and inter-investigator reliability for MSI and MSV were comparable to that for maximum intervertebral rotations as established in previous studies [7–9].

### MSI

Our study suggests that investigator image registration has a minimal influence on estimates of MSI during passive recumbent motion. The reported SEMs for intra- and inter-investigator repeatability for MSI in our study account for a small percentage of the group means of MSI during passive recumbent motion. These findings suggest that MSI derived from passive recumbent spine flexion may be a reliable measurement tool. Specifically, MSI measured in the passive recumbent position has been demonstrated to be greater in individuals with CNSLBP compared to healthy controls [5, 6], as well as in those with treatment-resistant LBP (i.e. previously treated with conservative therapy, surgery, or other interventional procedures). MSI has also been correlated with composite disc degeneration in a population with CNSLBP during passive recumbent motion, suggesting that an inequality of restraint in the passive subsystem (e.g. intervertebral discs, ligaments, facet joints) may be one mechanical factor linking disc degeneration to CNSLBP [5]. These findings contribute to the construct validity for MSI in passive recumbent motion and suggest a possible association between MSI and pain; however, the mechanisms for this are currently unknown. Given the established construct validity, substantial intra- and inter-investigator reliability, low SEMs, and moderate intrasubject reliability for MSI in a healthy population during passive recumbent lumbar spine flexion, MSI may be considered to be a valid and reliable biomechanical composite measure of multi-level intervertebral motion. Further work investigating the reliability of MSI in individuals with CNSLBP is warranted, particularly if there is potential use of MSI in clinical settings. However, a greater understanding of the role of increased MSI in CNSLBP is required (i.e. why it is a biomarker) before it can be routinely used to inform clinical management. QF is an advanced technology requiring special skills and continuous quality assurance procedures, making it most suitable as a specialist referral service, rather than a modality for routine use in practice premises. Although radiation exposure is considerably less than that of a standard lumbar spine radiographic examination, given our current level of understanding, risk-benefit to patients would not warrant routine use at this time. In the authors’ experience, referrals to a QF service are usually to investigate potential segmental instability in patients with CNSLBP, where results often reveal significant abnormal MSI values. Future studies should explore the threshold for how such results affect patient management decisions.

### MSV

In contrast to MSI, MSV had weaker inter- and intra-investigator repeatability during recumbent examinations, which may be related to its low values (mean 0.07) compared to MSI (0.24). In addition, MSV has been shown not to discriminate CNSLBP patients from controls in this configuration [5]. However, in standing flexion, MSV has been found to have considerably higher average values than in recumbent motion (0.17 compared with 0.08), making for potentially better repeatability in such studies. In weight bearing studies, it has also been found to be strongly associated with disc degeneration (*r* = 0.85), albeit in patients only, suggesting that it does have a role in diagnostic understanding [5]. Subsequent weight bearing flexion studies have found that neither MSI nor MSV discriminates patients from controls in this configuration [22]. However, the variability of proportional motion at the L4–5 level alone was found to be significantly higher in patients. This suggests that it would be worthwhile to repeat the present study in the weight bearing configuration, extending the analysis to individual levels.

### Limitations and further work

This study analysed MSI and MSV measured from passive recumbent flexion in a population of healthy individuals. Therefore, the repeatability results may not reflect the repeatability for active weight-bearing motion or the reliability in a population with CNSLBP. As the investigators involved in image analysis were the main subjects of interest in this study, we do not feel that repeatability estimates from a population with CNSLBP will be very different from the results of our study. According to previously published QF protocols, all participants (healthy controls and those with CNSLBP) had to have a body-mass index of less than 30 and be between the ages of 18 and 70. The current study only examined error that may have occurred from investigator input during the image analysis stage. Error from repeated measures of a subject reflecting their trial-to-trial variability were not taken into account. Although a previous study established intrasubject repeatability [10], determining the relative contribution of error associated with investigator input and error associated with the subject’s variability to the total measurement error remains a challenge. Future studies should evaluate other sources of error that may occur during QF image acquisition and analysis (e.g. intra- and inter-fluoroscope operator variability from image acquisition). This study also did not assess the effect of differences in training levels for image processing and analysis between the two investigators, and it is currently unknown whether training level affects the repeatability results. Future research should also establish repeatability estimates for MSI and MSV, as well as individual level proportional motion variability.in active weight-bearing motion and in symptomatic populations.

## Conclusion

Repeatability for intervertebral motion sharing during passive recumbent motion, specifically related to the effect of investigator analytical input during image analysis, was determined for passive recumbent flexion in a healthy population. MSI demonstrated substantial intra- and inter-investigator repeatability, suggesting that investigator analytical input has a minimal influence on the measurement. MSV demonstrated moderate intra-investigator reliability and fair inter-investigator repeatability. Confirmation in patients with CNSLBP is now required.

## Data Availability

The datasets used during the current study are available from the corresponding author on reasonable request.
